# Notes on Preparations for the Sick

**Published:** 1890-06

**Authors:** 


### Notes on Preparations for the Sick.

---

**Kolatina.**—T. Christy & Co., London.

The kola nut does not appear to be as well known as it deserves. In comparison with coffee, tea, and cocoa, it takes the first place as regards the amount of nitrogenous matter chemically defined and crystallisable. The amount of caffeine is 2.348 per cent., whereas coffee contains only 2.25. It appears to have the stimulating qualities of tea and coffee, but does not give rise to any digestive disturbance; it may therefore be taken by those who are unable to take either tea or coffee, and will be found to serve as a palatable and efficient substitute.

---

**Liquor Ouabain.**—T. Christy & Co., London.

Each drachm contains one-fifth of a milligramme, and this dose may be given three or four times daily.

Ouabain is the active principle of an arrow-poison used by the natives of Obock. It acts powerfully on the respiratory centres. Prof. M. E. Gley, of Paris, found that the principal effects of $\frac{1}{16}$th of a grain of ouabain on a dog were: marked slowing of the respirations, without much cardiac disturbance, lasting for a few minutes after the injection, and then ceasing. Larger doses first quickened respiration and then caused it gradually to get slower, until it ceased completely, the heart's pulsations continuing for several minutes afterwards. In the case where artificial respiration was used, the respirations were maintained as well as the heart's beats.

It has been suggested that ouabain would prove useful in asthma, pertussis, and heart disease (see *Medical Annual*, 1889, p. 56); and on this subject Prof. Gley writes: "I do not know of the use of ouabain in asthma and pertussis, but from my physiological experience it might prove to act favourably in asthma."

Like strophanthine, it has the property of arresting the frog's heart: the injection of even so small a quantity of crystallised ouabain as $\frac{1}{80}$th of a milligramme stops the heart in eight or nine minutes.

We have used ouabain in a few cases of pertussis with gratifying results, but our experience of this drug is mainly in cardiac affections. Ouabain is a pure heart-tonic, and does not appear to affect arterial tension. It is therefore indicated when we desire to stimulate the heart without raising the general arterial tension. In certain cases of organic heart disease attended with cardiac neuralgia, the relief was most marked and very quickly obtained. Ouabain may be given with advantage for functional palpitation of the heart, associated with dyspepsia.

The extremely poisonous properties of this drug must be borne in mind in prescribing it. For this reason only the active principle should be used, as in the standardised preparation of Messrs. Christy & Co.

---

**Salt Regal.**—THE SALT REGAL COMPANY, Liverpool.

The demand for salines, fed by the constant advertising of the same, never flags. It has recently been said that the English are a nation of pill-takers: it may equally be said that they revel in salines. If they must take something, we prefer the saline to the pill as being more pleasant and less harmful. Whether salines and longevity are necessarily associated as cause and effect may be open to question; but certainly these salines, if poisons, are very slow in action. But there appear to be fashions in salines as in most earthly matters; and the newest fashion is to combine a little antiseptic with a palatable effervescent, giving an ingenious combination, in the form of a white powder, which when mixed with water effervesces freely, and assumes the tint of a weak permanganate solution. It is an elegant preparation, pleasing to the fancy as well as to the eye, and withal thoroughly efficient as an aperient.

---

**Toilet Lanoline.**—BURROUGHS, WELLCOME, & CO., London.

Lanoline has decidedly established itself as a thing which means to stay. This particular form of it is an emollient, soothing application for irritable skins, useful for chapped lips and hands, abrasions, and eruptions of many kinds. In a thin layer over a wound it forms an efficient protective, as it has been shown by Professor Gottstein that such a layer of lanoline forms an impassable barrier to disease-germs. The preparation is elegantly perfumed, and conveniently mounted in collapsible tubes.

---

**Vinolia Soap.**—BLONDEAU & CIE., London.

Vinolia appears to have established for itself a wide reputation and great range of utility; and the Vinolia Soap, superfatted, is one of its most useful combinations. An analysis of this soap gives the following result:

| | |
|---|---|
| Free alkali ... ... ... ... | 0.00 |
| Combined alkali ... ... ... | 9.17 |
| Fatty anhydrides ... ... ... | 74.51 |
| Free fat ... ... ... ... ... | 3.81 |
| Scent, water, &c. ... ... ... | 12.51 |
| | 100.00 |

The irritating effects of many ordinary soaps on sensitive skins are well recognised, and hence the necessity for caution against the use of any soap in inflammatory forms of skin disease. Such a caution appears to be unnecessary if a superfatted soap like the above-mentioned be employed. The entire absence of excess of alkali, and the presence of unsaponified cream, prevent any deleterious action upon the skin. It is the most perfect soap we have ever had the pleasure to employ, and may safely be used where other soaps would be injurious.

